# Ca^2+^ in Hybridization Solutions for Fluorescence *in situ* Hybridization Facilitates the Detection of *Enterobacteriaceae*

**DOI:** 10.1264/jsme2.ME16186

**Published:** 2017-05-18

**Authors:** Shin Haruta, Takao Iino, Moriya Ohkuma, Ken-ichiro Suzuki, Yasuo Igarashi

**Affiliations:** 1 Department of Biological Sciences, Tokyo Metropolitan UniversityTokyoJapan; 2 Graduate School of Agricultural and Life Sciences, The University of TokyoTokyoJapan; 3 Japan Collection of Microorganisms, RIKEN BioResource CenterIbarakiJapan; 4 NITE Biological Resource Center (NBRC), National Institute of Technology and Evaluation (NITE)ChibaJapan; 5 Research Center of Bioenergy and Bioremediation, Southwest UniversityChongqingChina

**Keywords:** fluorescence *in situ* hybridization, *Enterobacteriaceae*, calcium ions, flow cytometry, *Escherichia coli*

## Abstract

Fluorescence *in situ* hybridization (FISH) has been employed to identify microorganisms at the single cell level under a microscope. Extensive efforts have been made to improve and extend the FISH technique; however, the development of a widely applicable protocol is a continuing challenge. The present study evaluated the effects of divalent cations in the hybridization solution on the FISH-based detection of various species of bacteria and archaea with rRNA-targeted probes. A flow cytometric analysis after FISH with a standard hybridization buffer detected positive signals from less than 30% of *Escherichia coli* IAM 1264 cells. However, the number of cells with positive signals increased to more than 90% after the addition of calcium chloride to the hybridization buffer. Mn^2+^ also had positive effects, whereas Mg^2+^ did not. The positive effects of Ca^2+^ were similarly observed for bacteria belonging to *Enterobacteriaceae*, including *Enterobacter sakazakii* IAM 12660^T^, *E. aerogenes* IAM 12348, *Klebsiella planticola* IAM 14202, and *Salmonella enterica* subsp. *enterica* serovar Typhimurium strain LT2. These results indicate that the supplementation of Ca^2+^ to the hybridization buffer for FISH contributes to the efficient detection of *Enterobacteriaceae* cells.

Fluorescence *in situ* hybridization (FISH) was developed to quantitatively detect a specific group of microbes, including those yet to be cultivated, by microscopy (1, 3). FISH is currently one of the key techniques utilized in microbial ecology and environmental microbiology (24, 30, 41) as well as in public health (*e.g.*, 20). FISH-based studies on microbiomes in humans and plants have recently attracted a great deal of attention (*e.g.*, 4, 7, 16, 36). A large number of probes that target rRNA have been designed (14, 42), and a standard protocol is available (https://www.arb-silva.de/). FISH protocols continue to be modified to increase the intensity of fluorescence and expand its application to the targeting of genomic DNA and mRNA (5, 19, 22, 23, 31, 43, 44). Recent advances in rRNA-targeted FISH combined with other spectrometric imaging techniques have resulted in a clearer visualization of the microbial world (18, 27, 37, 39).

rRNA-targeted FISH occasionally fails to detect microbial cells even if rRNA gene sequence and oligonucleotide probes match completely. Previous methodological studies achieved improvements in the hybridization efficiency of probes to their targets (12, 13, 32, 45, 47). Cell wall structures also affect FISH results. Extensive efforts have been made to increase the probe permeability of microbial cells (6, 8–10, 28, 34). Nevertheless, an acceptable fluorescent signal intensity is not always obtained from all cells, even in laboratory-grown pure cultures. This phenomenon was traditionally considered to be due to a low cellular ribosome content (11, 26, 40). In the present study, we demonstrated that this phenomenon was a significant cause for concern, particularly when attempting to detect bacteria in the family *Enterobacteriaceae*, which includes medically important species. However, this issue was resolved with a slight modification to the standard FISH protocol, *i.e.*, the addition of Ca^2+^ or Mn^2+^ to the hybridization solution.

## Materials and Methods

### Microbial strains and preparation of cells for FISH

Microorganisms (listed in Table 1) were obtained from public culture collections and cultivated according to their instructions. Cells were fixed with 3% paraformaldehyde pH 7.4, at 4°C for 20–24 h and stored in 99% ethanol:phosphate-buffered saline (PBS) solution (1:1 [v/v]) at −20°C as described by Wallner *et al.* (40). Prior to *in situ* hybridization, when indicated, the pretreatment of cells was conducted by an incubation in one of three solutions: 10 mg mL^−1^ of lysozyme at 37°C for 1 h, ethanol:formalin (9:1 [v/v]) at room temperature for 10 min, or 0.25 M HCl at room temperature for 30 min (8, 41).

### FISH and flow cytometry

FISH and flow cytometry were performed according to Amann *et al.* (2), Fuchs *et al.* (12), Manz *et al.* (25), and Yilmaz and Noguera (45). Fixed cells were suspended in standard hybridization buffer (0.9 M NaCl, 0.01% SDS, 20 mM Tris-HCl, pH 7.2 and 15% formamide) containing a fluorescein isothiocyanate (FITC)-labeled probe (EUB338) (1) or Alexa488-labeled probe (ARC915) (35) (0.5 pmol μL^−1^), as reported previously (1, 17, 35). In addition, the fluorescent probes, EC1153, EC1482, EC839S, EC839L, and EC1235, all of which have distinct target sequences (*Escherichia coli* 16S rRNA gene positions 1153–1172, 1482–1499, 839–856, 839–859, and 1235–1255, respectively) (12, 46), were used in experiments with *E. coli*. As a negative control, the fluorescent probe, non-EUB, which has a sequence complementary to that of EUB338, was used (48).

As supplemental cations, CaCl_2_, MnSO_4_, or MgCl_2_ was added to the hybridization buffer at appropriate concentrations. After incubating at 46°C for 20–24 h, the buffer was replaced with a washing solution (375 mM NaCl, 0.01% SDS, 20 mM Tris-HCl at pH 7.2 and 5 mM EDTA), and the sample was incubated at 46°C for 20 min. Cells were then suspended in PBS and analyzed on the same day. Fluorescently hybridized cells were analyzed with the flow cytometer, Epics Altra (Beckman Coulter, Miami, FL, USA). The experimental conditions used for flow cytometry included deionized particle-free water (Milli-Q water filtered through a membrane with a pore size of 0.1 μm) as sheath fluid and an argon laser at a wavelength of 488 nm (power level, 15 mW). Side scattering with a 488-nm band pass filter was used for the discrimination of bacterial cells from other particles. The green fluorescence of FITC and Alexa488 was assessed using the PMT2 channel (515 to 525 nm). EXPO32 (Beckman Coulter) was used for data analyses. A total of 50,000 events were analyzed in each sample at a data rate of <3,000 events s^−1^. The threshold for positive fluorescence was defined as a fluorescence signal intensity of less than 5% of the total events observed in the negative control (*i.e.*, without a fluorescent probe). Epifluorescence microscopic observations were performed after samples had been stained with 4′,6-diamidino-2-phenylindole (DAPI) using Axioskop2 (Carl Zeiss, Jena, Germany).

## Results and Discussion

### Effects of divalent cations on FISH for *E. coli* detection

**Fig. 1:**
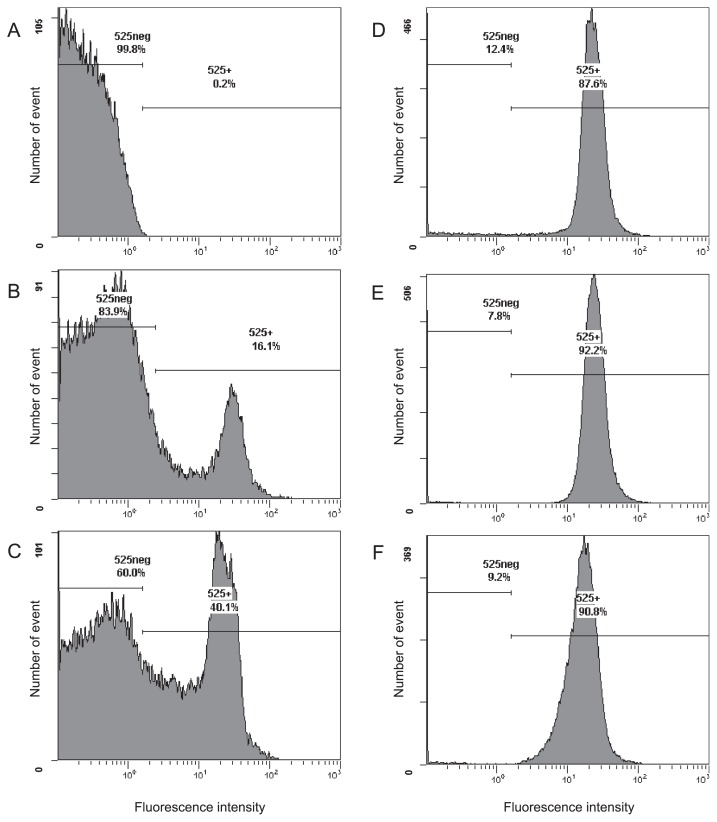
The distribution of the fluorescence signal intensity of cells hybridized in hybridization buffer supplemented with CaCl_2_. *E. coli* IAM 1264 cells were harvested at the stationary phase of growth and fixed with paraformaldehyde, as described in the Materials and Methods section. After *in situ* hybridization with the FITC-labeled probe, cells were analyzed with a flow cytometer. The x-axis and y-axis represent the fluorescence intensity (at 525 nm) and number of detected events, respectively. A, negative control (without probe); B, no divalent cation supplementation; C, 0.5 mg L^−1^ CaCl_2_; D, 5 mg L^−1^ CaCl_2_; E, 50 mg L^−1^ CaCl_2_; and F, 500 mg L^−1^ CaCl_2_. 525 neg, fraction of negative signals and 525+, fraction of positive signals.

Following standard protocols, *E. coli* IAM 1264 cells harvested at the stationary phase of growth were fixed with paraformaldehyde, and hybridized with the fluorescent-labeled probe EUB338 for 20 h (2, 12). Flow cytometry results showed that 16.1% of all cells exhibited a positive signal, *i.e.*, 83.9% of cells showed low fluorescence signal intensity (Fig. 1B; the positive signal percentage was 13.5±2.1% in three independent experiments using different batches of *E. coli* cultures), indistinguishable from cells incubated without the fluorescent probe (Fig. 1A). This low positive signal percentage may be caused by a decrease in the membrane permeability of cells in the stationary phase (21). The positive signal percentage was slightly increased by the addition of 0.5 mg L^−1^ of calcium chloride into the hybridization buffer (Fig. 1C) and markedly increased to more than 90% following the addition of 5 mg L^−1^ or more of calcium chloride (Fig. 1D, E, and F). A microscopic analysis of the hybridized cells also revealed that the ratio of fluorescently-labeled cells to total cells was clearly increased by the addition of calcium chloride (Fig. S1). As shown in Fig. 1D, E, and F, the fluorescence signal intensity from cells was not markedly affected by Ca^2+^. A similar effect of calcium ions was observed in *E. coli* IAM 1264 cells under multiple conditions; harvested in the exponential phase of growth, fixed with paraformaldehyde for a shorter time (4–6 h), and hybridized with EC1153, EC1482, EC839S, EC839L, and EC1235 probes targeting 16S rRNA (12, 46) (data not shown).

The effects of other divalent cations were evaluated (Fig. S2). Although supplementation with 50 mg L^−1^ of manganese sulfate produced no effect (Fig. S2A), the addition of 500 mg L^−1^ of manganese sulfate to the hybridization buffer mildly increased the ratio of hybridized cells (Fig. S2B). In contrast, supplementation with magnesium chloride reduced the number of positive events (Fig. S2C and D).

Divalent cations have stabilizing effects on the hybridization of nucleotides. However, the low concentrations of divalent cations used in this study were not expected to increase hybridization efficiency and reduce probe specificity in FISH. It has been theoretically and experimentally demonstrated that less than 0.5 mM of divalent cations (*i.e.*, below the effective concentrations of Ca^2+^ and Mn^2+^ in this study) has a negligible effect on the melting temperatures (*T*_m_) of oligonucleotides in the presence of high concentrations of Na^+^ greater than 0.1 M (29, 38). We confirmed that the supplemental addition of calcium chloride did not cause false positive signals when the probe non-EUB was applied as a negative control (data not shown).

Hanahan reported that divalent cations had a marked effect on the genetic transformation efficiency of *E. coli* by plasmids, and Ca^2+^ and Mn^2+^ were found to be more effective than Mg^2+^ (15). Hanahan also suggested that divalent cations affected the phosphate moieties of DNA, lipid bilayers, and lipopolysaccharides, resulting in an increase in the permeability of *E. coli* cells to oligonucleotide probes (15).

The pretreatment of cells prior to FISH has been shown to increase probe permeability (3, 34). Ootsubo *et al.* treated fixed enteric bacterial cells including *E. coli*, with a non-ionic detergent before hybridization (33). In the present study, we found that positive signal percentages after ethanol:formalin and HCl treatments of *E. coli* IAM 1264 cells were 59.8% and 26.9%, respectively, and the lysozyme treatment was as effective (the number of positive signals out of all events, 93.8%) as the addition of Ca^2+^ to the hybridization buffer. However, the supplementation of Ca^2+^ to the hybridization buffer is a simple solution that does not alter cell morphology for the efficient detection of *E. coli*.

### Effects of Ca^2+^ on FISH detection of a number of species of bacteria and archaea

The effects of Ca^2+^ on additional bacterial and archaeal species were evaluated. FISH and flow cytometry were completed on 27 species of bacteria and two species of archaea using the EUB338 probe for bacteria and the ARC915 probe for archaea. The ratio of positive events to the total number of events was assessed for each species using cells harvested at the late exponential phase of growth (Table 1). As observed in *E. coli* IAM 1264, the ratio of positive cells in *E. coli* IAM 12119^T^ was low under standard conditions; however, the number of positive cells increased after the addition of calcium chloride to the hybridization buffer. Similarly, a low positive signal ratio was observed for *Enterobacter* spp., *Klebsiella planticola* IAM 14202^T^, *Citrobacter freundii* NBRC 1268^T^, and *Salmonella enterica* subsp. *enterica* serovar Typhimurium strain LT2 in the family *Enterobacteriaceae* in *Gammaproteobacteria*; however, the supplementation of calcium chloride to the hybridization buffer increased the number of positive cells. Other species in *Enterobacteriaceae* showed a relatively high percentage of positive cells under standard conditions, such as *Proteus vulgaris* JCM 1668^T^, *Pantoea agglomerans* IAM 12659, and *K. pneumoniae* IAM 14200^T^, and the effects of calcium chloride were still positive (Table 1). However, as shown in Table 1, calcium chloride did not markedly alter the FISH results obtained for bacterial and archaeal species other than those for *Enterobacteriaceae*.

## Conclusions

The addition of Ca^2+^ to the hybridization buffer for FISH increased the number of positive signals from *E. coli*. This effect was independent of the probe sequence and appears to have been caused by an increase in membrane permeability for oligonucleotide probes. Ca^2+^ was similarly effective for bacteria in *Enterobacteriaceae* including *S. enterica* subsp. *enterica* serovar Typhimurium strain LT2. These *Enterobacteriaceae* cells may have a distinctive cell wall structure. Calcium chloride at a concentration of less than 50 mg L^−1^ did not produce negative effects in any of the microbes examined in this study (Table 1). As a simple application of our results, bottled hard water (>20 mg L^−1^ of Ca^2+^ and <100 mg L^−1^ of Mg^2+^) may be used to prepare hybridization solutions rather than the pure water typically used in laboratory settings. The protocol introduced in this study is useful for analyzing intestinal bacterial flora and detecting pathogenic bacteria including *E. coli* and *Salmonella* in medical and public health fields.

## Supplementary material



## Figures and Tables

**Table 1:** Summary of flow cytometry results after FISH

Tested strains	Ratio of positive events (%)*	

	w/Ca^2+^	w/o Ca^2+^
*Gammaproteobacteria (Enterobacteriaceae)*		
*Escherichia coli* IAM 1264	92.7	28.6
*Escherichia coli* IAM 12119^T^	66.7	5.0
*Proteus vulgaris* JCM 1668^T^	90.1	76.5
*Pantoea agglomerans* IAM 12659	68.9	58.1
*Enterobacter sakazakii* IAM 12660^T^	68.9	10.9
*Enterobacter aerogenes* IAM 12348^T^	78.0	11.4
*Klebsiella planticola* IAM 14202^T^	90.3	41.4
*Klebsiella pneumoniae* IAM 14200^T^	95.9	58.5
*Citrobacter freundii* NBRC 1268^T^	39.0	9.3
*Salmonella enterica*		
subsp. *enterica* serovar Typhimurium LT2	80.1	38.4
*Gammaproteobacteria* (others)		
*Pseudomonas aeruginosa* JCM 14847	90.6	90.0
*Aeromonas salmonicida* NBRC 13784^T^	47.8	47.1
*Xanthomonas pisi* NBRC 13556	33.6	25.9
*Vibrio orientalis* NBRC 15638^T^	96.5	96.3
*Shewanella putrefaciens* NBRC 3908^T^	92.7	93.6
*Alphaproteobacteria*		
*Acetobacter pasteurianus* IAM 1804	21.2	25.1
*Betaproteobacteria*		
*Comamonas testosteroni* NBRC 14951^T^	87.2	76.6
*Chromobacterium violaceum* NBRC 12614^T^	86.5	86.2
*Firmicutes*		
*Bacillus subtilis* IAM 12118^T^**	92.9	93.9
*Bacillus licheniformis* IAM 13417**	94.5	93.9
*Clostridium acetobutylicum* NBRC 13948^T^**	84.3	84.3
*Staphylococcus aureus* IAM 1058**	77.1	81.7
*Lactobacillus plantarum* JCM 1149	63.9	62.7
*Lactococcuss lactis* subsp. *lactis* IAM 1249	62.8	65.2
*Bacteroidetes*		
*Bacteroides vulgatus* NBRC 14291^T^	95.8	94.8
*Actinobacteria*		
*Bifidobacterium bifidum* NBRC 100015^T^	50.0	51.1
*Corynebacterium glutamicum* NBRC 12168^T^**	59.4	59.6
*Micrococcus luteus* ATCC 398**	68.0	68.1
*Euryarchaeota*		
*Methanococcus voltae* NBRC 100457^T^	96.2	95.4
*Crenarchaeota*		
*Acidianus brierleyi* DSM 1651	56.9	58.0

Cells were harvested at the late exponential phase of growth and fixed with paraformaldehyde, as described in the Materials and Methods section.

* The threshold for positive fluorescence was defined by <5% of total events observed in the negative control (*i.e.*, without probe). Positive events % were assessed after FISH with the hybridization buffer containing 50 mg L^−1^ of calcium chloride (w/Ca^2+^) and not containing calcium chloride (w/o Ca^2+^). Average values from at least two independent experiments are shown.

** The lysozyme treatment was conducted for these bacterial species before hybridization.
