# Supplementary material for: Ca2+ in Hybridization Solutions for Fluorescence in situ Hybridization Facilitates the Detection of Enterobacteriaceae
Source: Microbes Environ. 2017 May 18;32(2):142–6. doi: 10.1264/jsme2.ME16186 (PMC5478537; doi:10.1264/jsme2.ME16186)
Supplement: Supplementary file 1 [file 32_142_s1.pdf]

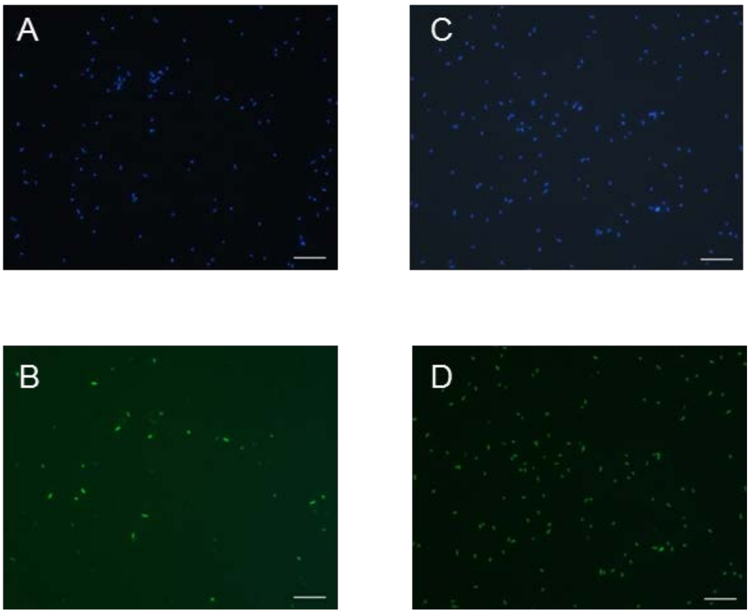

Fig. S1. Epifluorescence micrographs of *E. coli* IAM 1264 cells hybridized in a hybridization buffer supplemented with no $CaCl_2$ (A, B) and 500 mg L^{-1} $CaCl_2$ (C, D). FISH was performed as shown in Fig. 1. After *in situ* hybridization with the FITC-labeled probe, the cells were stained with DAPI. Images captured by using the DAPI filter set (A, C) and the fluorescein filter set (B, D) are shown for identical fields. Bars, 20 µm.

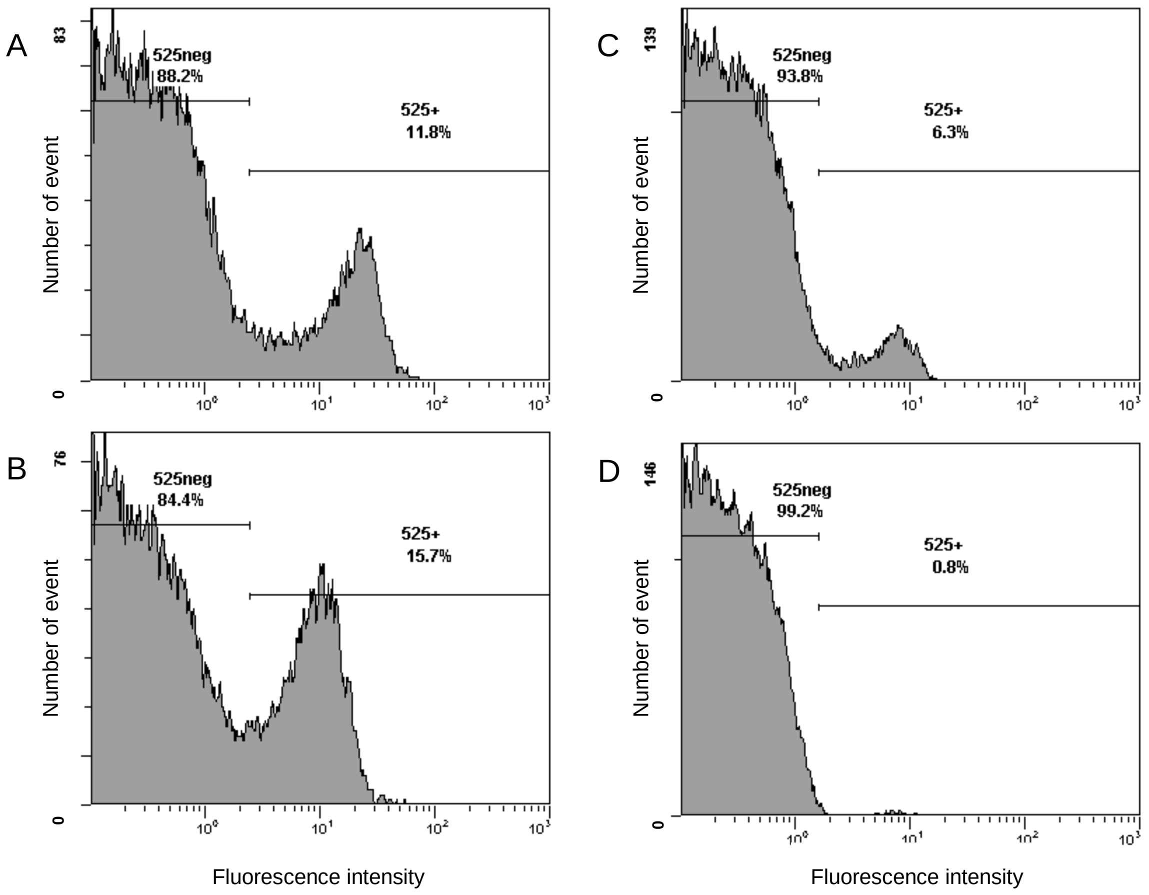

Fig. S2. The distribution of fluorescence signal intensity of cells hybridized in a hybridization buffer supplemented with $MnSO_4$ or $MgCl_2$. *E. coli* IAM 1264 cells were harvested at the stationary phase of growth and fixed with paraformaldehyde as described in the Materials and Methods section. After *in situ* hybridization with the FITC-labeled probe, the cells were analyzed with flow cytometer as shown in Fig. 1. The x-axis and y-axis represent the fluorescence intensity (at 525 nm) and number of detected events, respectively. A, 50 mg L^{-1} $MnSO_4$; B, 500 mg L^{-1} $MnSO_4$; C, 50 mg L^{-1} $MgCl_2$; and D, 500 mg L^{-1} $MgCl_2$. 525neg, fraction of negative signals and 525+, fraction of positive signals.
